# Extensive characterization of *Campylobacter jejuni* chicken isolates to uncover genes involved in the ability to compete for gut colonization

**DOI:** 10.1186/s12866-015-0433-5

**Published:** 2015-05-10

**Authors:** Alexandre Thibodeau, Philippe Fravalo, Eduardo N. Taboada, Sylvette Laurent-Lewandowski, Evelyne Guévremont, Sylvain Quessy, Ann Letellier

**Affiliations:** Department of Pathology and Microbiology, NSERC Industrial Research Chair in Meat-Safety (CRSV), University of Montreal, Veterinary Medicine Faculty, Saint-Hyacinthe, QC Canada; Department of Pathology and Microbiology, Swine and Avian Infectious Disease Research Centre (CRIPA), University of Montreal, Veterinary Medicine Faculty, Saint-Hyacinthe, QC Canada; Department of Pathology and Microbiology, Groupe de recherche et d’enseignement en salubrité alimentaire (GRESA), University of Montreal, Veterinary Medicine Faculty, Saint-Hyacinthe, QC Canada; Agriculture and Agri-Food Canada, Food Research and Development Centre, St-Hyacinthe, QC Canada; Public Health Agency of Canada, Laboratory for Foodborne Zoonoses, Lethbridge, AB Canada

**Keywords:** *Campylobacter jejuni*, Chicken, Colonization, CGF typing, Autoagglutination, Chemotaxis, Adhesion and invasion, Competition

## Abstract

**Background:**

*Campylobacter jejuni* is responsible for human foodborne enteritis. This bacterium is a remarkable colonizer of the chicken gut, with some strains outcompeting others for colonization. To better understand this phenomenon, the objective of this study was to extensively characterize the phenotypic performance of *C. jejuni* chicken strains and associate their gut colonizing ability with specific genes.

**Results:**

*C. jejuni* isolates (*n* = 45) previously analyzed for the presence of chicken colonization associated genes were further characterized for phenotypic properties influencing colonization: autoagglutination and chemotaxis as well as adhesion to and invasion of primary chicken caecal cells. This allowed strains to be ranked according to their *in vitro* performance. After their *in vitro* capacity to outcompete was demonstrated *in vivo*, strains were then typed by comparative genomic fingerprinting (CGF). *In vitro* phenotypical properties displayed a linear variability among the tested strains. Strains possessing higher scores for phenotypical properties were able to outcompete others during chicken colonization trials. When the gene content of strains was compared, some were associated with different phenotypical scores and thus with different outcompeting capacities. Use of CGF profiles showed an extensive genetic variability among the studied strains and suggested that the outcompeting capacity is not predictable by CGF profile.

**Conclusion:**

This study revealed a wide array of phenotypes present in *C. jejuni* strains, even though they were all recovered from chicken caecum. Each strain was classified according to its *in vitro* competitive potential and its capacity to compete for chicken gut colonization was associated with specific genes. This study also exposed the disparity existing between genetic typing and phenotypical behavior of *C. jejuni* strains.

**Electronic supplementary material:**

The online version of this article (doi:10.1186/s12866-015-0433-5) contains supplementary material, which is available to authorized users.

## Background

*Campylobacter jejuni* is the bacterial agent responsible for campylobacteriosis, a severe gastro-enteritis afflicting humans. Campylobacteriosis may be acquired via the consumption of contaminated food [1]. Poultry meat products are one of the most important vectors of *C. jejuni* to humans [2,3]. High numbers of *C. jejuni* can colonize the chicken caecum, up to 10^9^ CFU/g of caecal matter [4]. Despite being present in such quantities, the bacterium mostly causes no harm to its avian host [4]. The mechanisms employed by the bacteria to colonize the chicken intestine are still not fully understood. Moreover, it was demonstrated that different *C. jejuni* strains do not have the same ability to colonize a chicken, with some strains outcompeting others when colonizing chicken cecum [5,6].

Many studies have reported the importance of certain phenotypic properties for efficient *C. jejuni* chicken colonization [7]. Individually, it was reported that the autoagglutination [8], chemotaxis [7,9], adhesion [7], and invasion [10] properties of a given strain influence its capacity to colonize the chicken gut. Unfortunately, no information is available on the distribution, interaction, and relative importance of these properties in field strains. While several genes have been identified as involved in chicken colonization, many of these are unequally distributed or show sequence diversity among chicken strains [11]. Their contribution to the outcompeting capacity of a given strain has yet to be described [12].

The aim of this study was to extensively characterize multiple chicken isolated *C. jejuni* strains for phenotypical properties in order to examine the possible association between these characteristics and the ability of chicken *C. jejuni* strains to outcompete others during colonization. Based on a previous characterization of these strains regarding chicken associated colonization genes, we also evaluated the association between a strain outcompeting capacity and its gene content.

## Method

### Strains and media

Sampling and identification of the isolates were done as previously described [11] from chicken caecal content recovered at slaughterhouses. A total of 45 *C. jejuni* isolates were used in this study. Confirmed *C. jejuni* isolates were frozen in multiple aliquots at − 80°C in Brucella broth (Innovation Diagnostic Inc., Montreal, Canada) containing 0.1 % Agar (Innovation Diagnostic Inc.) and 25 % (*v/v*) glycerol. Strain *C. jejuni* 81–176, used as a positive control, was kindly supplied by Dr. Shaun Cawthraw, Veterinary Laboratories Agency, UK. Strains were cultured on mCCDA (Innovation Diagnostic Inc.), Mueller Hinton Agar (Oxoïd, Nepean, Ontario, Canada), or Trypticase Soy agar (TSA) supplemented with 5 % (*v/v*) sheep blood (Fisher Scientific, Ottawa, Ontario, Canada) and incubated in a microaerobic atmosphere using Oxoïd gas-generating system. For every characterization assay, a new − 80°C aliquot of the strain was used to minimize strain variation due to repeated *in vitro* passages.

### *In vitro* characterization

*C. jejuni* strains were characterized for their ability to autoagglutinate, be attracted by mucins, and adhere to and invade chicken primary caecal cells. Each result was the mean of at least two distinct experiments, each performed in technical duplicates. For each *in vitro* phenotypic characterization, strains were then classified according to their phenotypical properties; the strain possessing the lowest property value (poorest performing strain) was assigned a rank value of 1 and the best one a rank value of 45. The mean rank of the *in vitro* phenotypic properties (autoagglutination, chemotaxis, adhesion, and invasion) was then calculated for each strain in order to estimate the strain’s chicken competition potential for the colonization of the chicken gut. Strains were numbered according to their mean rank value classification and this number was used in the figures to identify each strain position according to their phenotypical characterization. Thus strain number 45, possessing the highest mean rank value, was expected to reveal a higher competition potential compared to all strains tested in this study (Table 1).

**Table 1:** *Campylobacter jejuni* strain global comparison

**Strain (Strain number)**	**Phenotypical properties**					**k-means partitioning**		
	**Adh**	**Inv**	**Chm**	**Agg**	**Mean**	***Group 1***	***Group 2***	***Group 3***
	**rank**	**rank**	**rank**	**rank**	**rank**			
D2008b (1)	1	1	14	10	7	+		
P2003a (2)	4	31	1	3	10	+		
F2008c (3)	10	12	9	13	11	+		
F2008a (4)	3	16	2	25	12	+		
B2008c ^1^ (5)	9	3	23	12	12	+		
M2003b (6)	23	7	19	6	14	+		
P2003b (7)	39	13	3	1	14	+		
O2003b (8)	6	27	11	15	15	+		
X2003 (9)	15	4	7	34	15		+	
E2008b (10)	8	8	16	29	15		+	
K2003a (11)	20	23	8	11	16	+		
T2003c (12)	5	44	4	9	16	+		
S2003a (13)	14	20	24	5	16	+		
U2003b (14)	13	6	44	7	18		+	
B2008b ^2^ (15)	12	22	25	14	18	+		
81-176 (16)	16	11	28	19	19		+	
B2008a (17)	19	5	21	30	19		+	
E2008c (18)	18	9	5	43	19		+	
T2003b (19)	17	14	10	37	20		+	
C2008a (20)	7	18	31	31	22		+	
D2008a ^3^ (21)	2	26	36	24	22		+	
W2003a (22)	24	2	43	20	22		+	
H2008a (23)	22	17	27	23	22		+	
I2008c (24)	33	29	34	2	25			+
R2003 (25)	31	38	15	17	25			+
C2008b (26)	26	10	30	36	26		+	
F2008b (27)	30	28	6	39	26			+
A2008c (28)	37	35	17	22	28			+
J2003a (29)	44	30	32	8	29			+
K2003b (30)	21	34	38	21	29			+
N2003b (31)	38	32	12	32	29			+
I2008b (32)	27	43	40	4	29			+
L2003b (33)	29	19	22	45	29		+	
N2003a (34)	43	25	20	28	29			+
Q2003a (35)	42	40	18	16	29			+
A2008d (36)	45	15	29	27	29			+
J2003b (37)	11	41	33	33	30			+
H2008c (38)	41	24	13	42	30			+
T2003a (39)	25	21	45	35	32		+	
O2003a (40)	35	42	39	18	34			+
F2008d ^3^ (41)	28	33	37	41	35			+
L2003a (42)	34	39	26	44	36			+
G2008b ^1^ (43)	32	45	42	26	36			+
G2008c (44)	36	36	35	40	37			+
A2008a ^2^ (45)	40	37	41	38	39			+

Strain identification begins with a letter (example **H**2008b) representing the lot origin, followed by the year (example H**2008**b) of sampling, and ends with an identification letter (example H2008**b**) to differentiate between strains isolated from the same lot; The strain number represents the strain ability to compete for chicken colonization with strain number 45 being the strain presenting the best out competing potential; Agg = autoagglutination; Chm = chemotaxis; Inv = invasion; Adh = adhesion; Strains underlined and sharing the same superscript were the strains co-inoculated in the chicken colonization competition assay.

### Autoagglutination

Autoagglutination and chemotaxis experiments were carried out as previously described [13]. For autoagglutination, overnight cultures were resuspended in PBS to an optic density (OD) (630 nm) of 1.0. One ml suspensions were incubated at room temperature for 3 h before the OD was remeasured. Autoagglutination was expressed as (Initial OD 630 nm − 3 h OD 630 nm)/Initial OD 630 nm X 100.

### Chemotaxis

For chemotaxis, overnight cultures were suspended to an OD of 1.0. Suspensions were mixed with an equal volume of soft agar (0.4 %). The resulting mix was then poured in a Transwell insert (BD, Mississauga, Ontario). The inserts were subsequently put in a 24-well plaque containing PBS (negative control) or porcine mucins (2 mg/ml) (Sigma-Aldrich, Oakville, Ontario, Canada) for 3 h. The number of bacteria found in the PBS or mucins conditions was then enumerated on Mueller-Hinton Agar. Chemotaxis results were described as follows: −1/log (number of recovered bacteria after the experiment/number of initial bacteria used).

### Adhesion and invasion of primary chicken caecal cells

Primary cells were obtained as follows, adapted from Byrne et al., 2007 [14]. Caeca collected at nearby slaughterhouses were emptied of their content, and, within an hour, transported to the laboratory in DMEM (Invitrogen) containing 1 % FBS (*v/v*) (Hyclone, Fisher Scientific), 200 units/ml of penicillin/streptomycin (Sigma-Aldrich), and 50 μg/ml of gentamicin (Sigma-Aldrich). After five washes in HBSS, caeca were cut into small pieces and digested using 375 U/ml of collagenase (Sigma-Aldrich) and 1 U/ml of dispase (Sigma-Aldrich) for 3 h at 37°C in DMEM supplemented with 1 % FBS (*v/v*), 200 units/ml of penicillin/streptomycin, and 50 μg/ml of gentamicin. Floating crypts were pelleted by centrifugation at 50 x *g* for 4 min. The cells were purified by suspending the pellet in HBSS containing 2 % D-Sorbitol (*v/v*) (Fischer Scientific) and then centrifuged at 50 x g for 4 min. After four purification steps, cells were seeded (3000 crypts per well) in 24-well cell culture plates (Fisher Scientific) using a culture media composed of DMEM supplemented with 10 % FBS (*v/v*), 1.4 μg/ml of hydrocortisone (Sigma-Aldrich), 10 μg/ml of insulin (Sigma-Aldrich), 5 μg/ml of transferrin (Sigma-Aldrich), 1 μg/ml of fibronectin (Sigma-Aldrich), 200 units/ml of penicillin/streptomycin, and 50 μg/ml of gentamicin. The culture was incubated at 38°C in a 6 % CO_2_ atmosphere. Half of the cell culture media was replaced every two days. After seven to nine days, cells (minimum 80 % confluence) were used for the characterization of *C. jejuni* isolates.

Ready to use primary caecal cells were first washed with Hank’s Balanced Salt Solution (HBSS) (Invitrogen, Burlington, Ontario, Canada) and then infected with 10^7^ CFU of *C. jejuni* per well. The infected cell culture was incubated for 3 h at 38°C and then washed three times with HBSS. Cells were lysed with PBS containing 0.5 % (*v/v*) Triton X-100 (Sigma-Aldrich) and the *C. jejuni* recovered were enumerated on Mueller-Hinton Agar. For invasion, prior to lysis, cells were further incubated for one hour in DMEM (Invitrogen) containing 1 % FBS (*v/v*) and 50 μg/ml of gentamicin, a concentration lethal for all tested isolates, and then washed in HBSS three times.

### Competition potential

K-means clustering was used (Gene Cluster 3.0) [15] to objectively separate the strains according their competition potential. For each strain, autoagglutination, chemotaxis, adhesion, and invasion ranks were defined as variables. The similarity metric used was the Euclidean distance, with 3 clusters and 1000 runs. A k of 3 was used as we wanted strains to split into a group of low, medium, or high competition potential, with strains showing clear opposite phenotypic properties. The resulting tree was visualized in Treeview [16].

### Chicken colonization and competition assays

All animal experiments were approved by the “Comité d’éthique sur l’utilisation des animaux” (CEUA) of the Veterinary Medicine Faculty of the University of Montreal. Day-old chickens (*n* = 9) were separated into three groups housed in separate pens. Birds were raised on wood shavings on concrete-floored pens and had *ad libitum* access to water and to a standard commercial feed. Just before the inoculation, fresh caecal droppings were collected to investigate for the presence of *C. jejuni*. At 14 days of age, each chicken was simultaneously inoculated *per os* with a mix suspension (1:1 ratio) of two different *C. jejuni* strains possessing opposed phenotypic properties (Table 1). All inoculum were equal: approximately 4 log CFU were used for each strain per bird. The three chickens in Group A received strains G2008b and B2008c; the three chickens in Group B received strains A2008a and B2008b; the three chickens in Group C received strains F2008d and D2008a. All chickens were euthanized in a CO_2_ chamber seven days later and the concentration of caecal *C. jejuni* was enumerated on mCCDA plates. The experience was repeated once.

### PCR detection of challenge strains

DNA was extracted using a standard phenol-chloroform procedure [17] from the caecal samples recovered seven days post-inoculation. The caecal samples represented the whole content of individual chicken caecal content (100 μL of 10^−3^ dilution) incubated 24 h on an mCCDA plate. The detection of genes specific to each inoculated strain was done by PCR (Table 2 and Table 3). The targeted genes were selected based on a prior microarray study [11]. DNA extracted from each inoculated strain served as controls. Standard mixes consisted of a 20 μl reaction containing 1X QPCR master mix (MBI EVOlution EvaGreen (R), Montréal Biotech Inc.) and 350 nm of each primer. Reactions that were positive after 35 cycles were rejected.

**Table 2:** PCR conditions for the detection of the genes specific to the strains used in the *in vivo* chicken colonization competition assay

**Gene**	**Primers (5′–3′)**	**Cycle**
*pse*D	F: GATGGAATGGTAAGCGTTGCACA	95°C for 20 s
	R: TTATCCTTGCTCCACCTTCGGTGC	56°C for 20 s
		72°C for 20 s
*vir*B11	F: TCTTGTGAGTTGCCTTACCCCTTTT	95°C for 20 s
	R: CCTGCGTGTCCTGTGTTATTTACCC	65°C for 20 s
		72°C for 20 s
CJ0144	F: TTCATGAAGTTGTGAATGCTGAAA	95°C for 20 s
	R: TCGCTTACAACAAGTTCGCC	61°C for 20 s
		72°C for 10 s

All PCR cycles were preceded by an initial denaturation step consisting of a 15-min incubation at 95°C; All PCR runs were ended by a final 5-min elongation step at 72°C followed by a denaturation curve analysis.

**Table 3:** Identification of the dominant *Campylobacter jejuni* strains in the *in vivo* chicken colonization competition assays by PCR detection of relevant genes

**Group and assay**	**Sample**	**Sample type**	***ars*** **C**	***vir*** **B11**	**cj0144**	***tet*** **O**	***rlo*** **B**	**cje0256**
Group1- assay1	Bird1	bird feces	+	-	+	+	+	-
Group1- assay1	Bird2	bird feces	+	-	+	+	+	-
Group1- assay1	Bird3	bird feces	+	-	+	+	+	-
Group1- assay2	Bird1	bird feces	+	-	+	+	+	-
Group1- assay2	Bird2	bird feces	+	-	+	+	+	-
Group1- assay2	Bird3	bird feces	+	-	+	+	+	-
Group1- assay1 + 2	G2008b	inoculated strain DNA	+	-	+	+	+	-
Group1- assay1 + 2	B2008c	inoculated strain DNA	-	+	-	-	-	-
Group2- assay1	Bird1	bird feces	+	-	+	-	+	-
Group2- assay1	Bird2	bird feces	+	-	+	-	+	-
Group2- assay1	Bird3	bird feces	+	-	+	-	+	-
Group2- assay2	Bird1	bird feces	+	-	+	-	+	-
Group2- assay2	Bird2	bird feces	+	-	+	-	+	-
Group2- assay2	Bird3	bird feces	+	-	+	-	+	-
Group2- assay1 + 2	A2008a	inoculated strain DNA	+	-	+	-	+	-
Group2- assay1 + 2	B2008b	inoculated strain DNA	-	+	-	-	-	-
Group3- assay1	Bird1	bird feces	-	-	+	+	-	-
Group3- assay1	Bird2	bird feces	-	-	+	+	-	-
Group3- assay1	Bird3	bird feces	-	-	+	+	-	-
Group3- assay2	Bird1	bird feces	+ (low)	-	+	+	-	-
Group3- assay2	Bird2	bird feces	+ (low)	-	+	+	-	-
Group3- assay2	Bird3	bird feces	-	-	+	+	-	-
Group3- assay1 + 2	F2008d	inoculated strain DNA	-	-	+	+	-	-
Group3- assay1 + 2	D2008a	inoculated strain DNA	-	-	-	+	-	+

Detection of genes specific to the inoculated strains in the *in vivo* competition assays seven days post-inoculation; (+) gene detected in the sample; (−) gene not detected in the sample; (low) weak signal observed.

### Gene association with phenotypic properties

A total of 30 strains, originating from different chicken lots or possessing different phenotypical properties, were retained for the comparison of their evaluated gene content [11] and the mean ranking of their phenotypic properties and thus their outcompeting capacity.

First, the genes known to be present in the strain (A2008a) possessing the highest outcompeting capacity were compared with those present in the strain (D2008b) possessing the lowest outcompeting capacity (Table 1). A list of all unique genes was established for both strains. Secondly, for each identified gene from all of the 30 retained strains, the mean rank value of the strains possessing a specific gene was compared to the mean rank value of the strains lacking this same gene. This allowed for the identification of genes that were present in strains that possessed, on average, higher or lower phenotypic mean rank values and thus high or low outcompeting capacities, as illustrated in the *in vivo* assay.

### Comparative genomic fingerprinting

To gain information about the representativity of our strain collection in Canada, all strains were subjected to CGF, a reference *Campylobacter* typing method, as previously described [18]. Briefly, DNA was extracted using Qiagen DNeasy Blood and Tissue kit (Qiagen, Toronto, Canada). The detection of 40 *C. jejuni* accessory genes was then carried out using a series of multiplex PCRs combined with QIAxcel automated electrophoresis for detection and analysis of the amplicons (Qiagen). The CGF profiles, which are based on the presence/absence of target genes, were clustered using a simple matching distance coefficient and the unweighted-pair group method using average linkages (UPGMA) of clustering in Bionumerics (v.6.1; Applied Maths, Austin, TX). CGF profiles were subsequently compared with those present in a Canadian CGF database comprising data on over 15,000 isolates from human, animal, and environmental sources (E. Taboada, personal communication).

### Statistical analysis

The Kruskal-Wallis test was used to compare the strains’ phenotypic properties and mean rankings. The comparison of the strains phenotypic mean ranking values, based on the presence or absence of certain genes, was done using the Mann–Whitney analysis. An alpha value lower than 0.05 was considered for genes present in strains likely to possess different mean rank values, while an alpha value lower than the alpha value divided by the number of compared genes was used to identify genes strongly associated with a strain’s outcompeting capacities. All statistical analyses were computed in GraphPad Prism 6 (GraphPad, La Jolla, CA, USA).

## Results

### Phenotypic properties of the strains

**Fig. 1:**
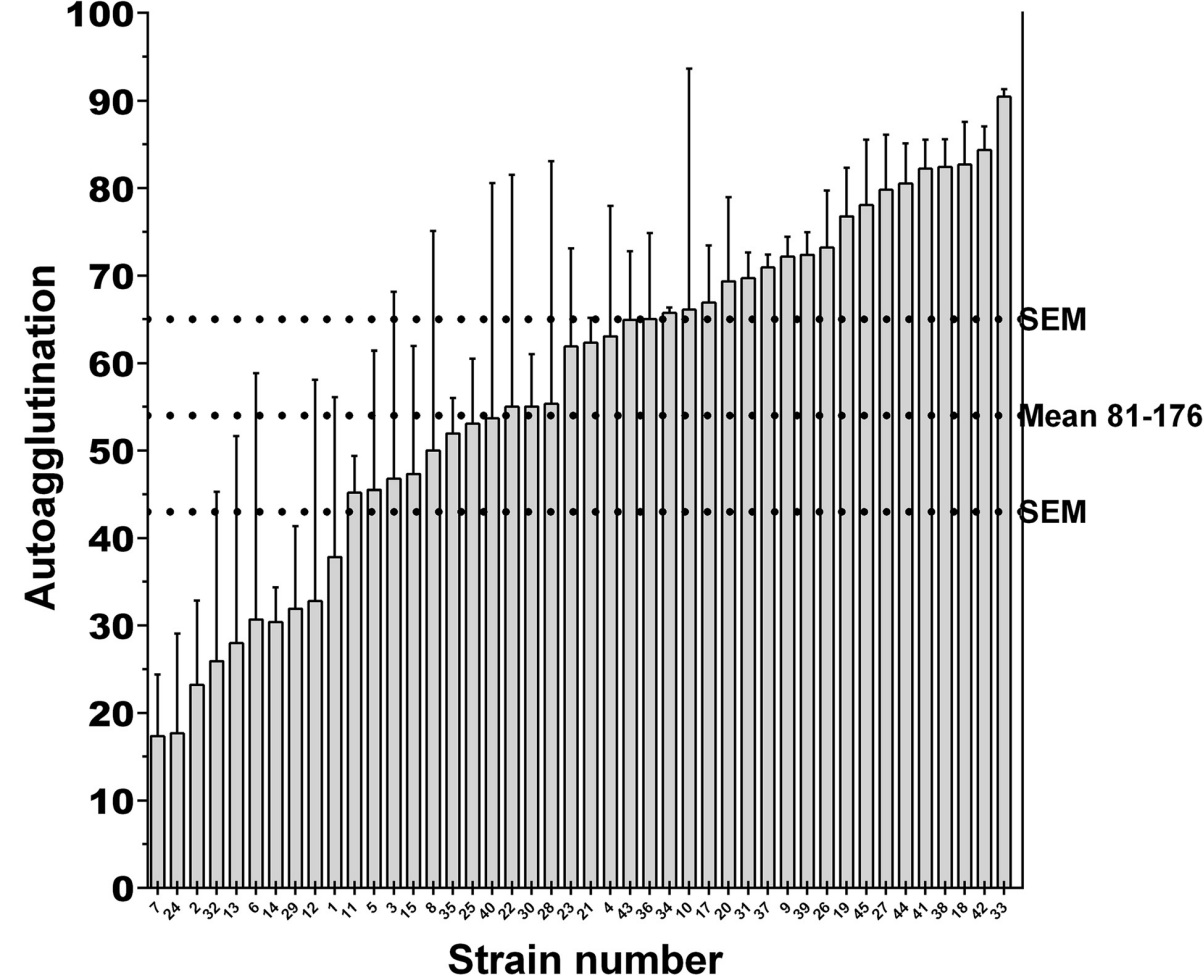
Autoagglutination capacities of *Campylobacter jejuni* chicken strains. The error bars represent the SEM. The horizontal dashed lines represent the control strain 81–176 and its SEM. Strain results were classified by increasing order. The x axis represents the strains numbered according to their final mean rank value, presented in Table 1. Strains were found with different autoagglutination properties (Kruskal-Wallis, *p* = 0.0083). Autoagglutination is expressed as ((Initial DO 630 nm – 3 h DO 630 nm)/Initial DO 630 nm) X 100

**Fig. 2:**
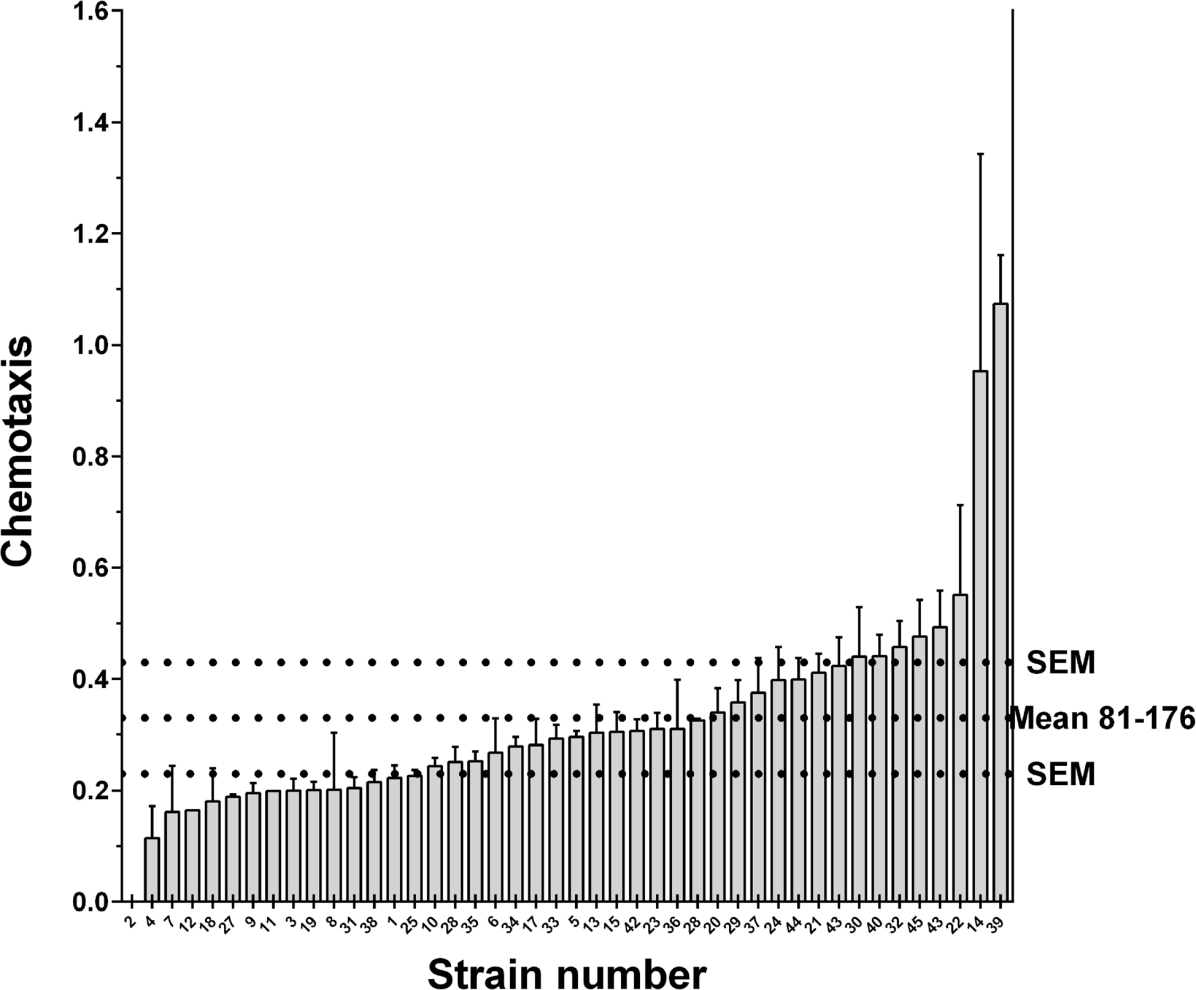
Chemotaxis properties of *Campylobacter jejuni* chicken strains. The error bars represent the SEM. The horizontal dashed lines represent the control strain 81–176 and its SEM. Strain results were classified by increasing order. The x axis represents the strains numbered according to their final mean rank value, presented in Table 1. Strains were found with different chemotaxis properties (Kruskal-Wallis, p < 0.0001). Chemotaxis is expressed as −1/log (attracted bacteria/initial bacteria used)

**Fig. 3:**
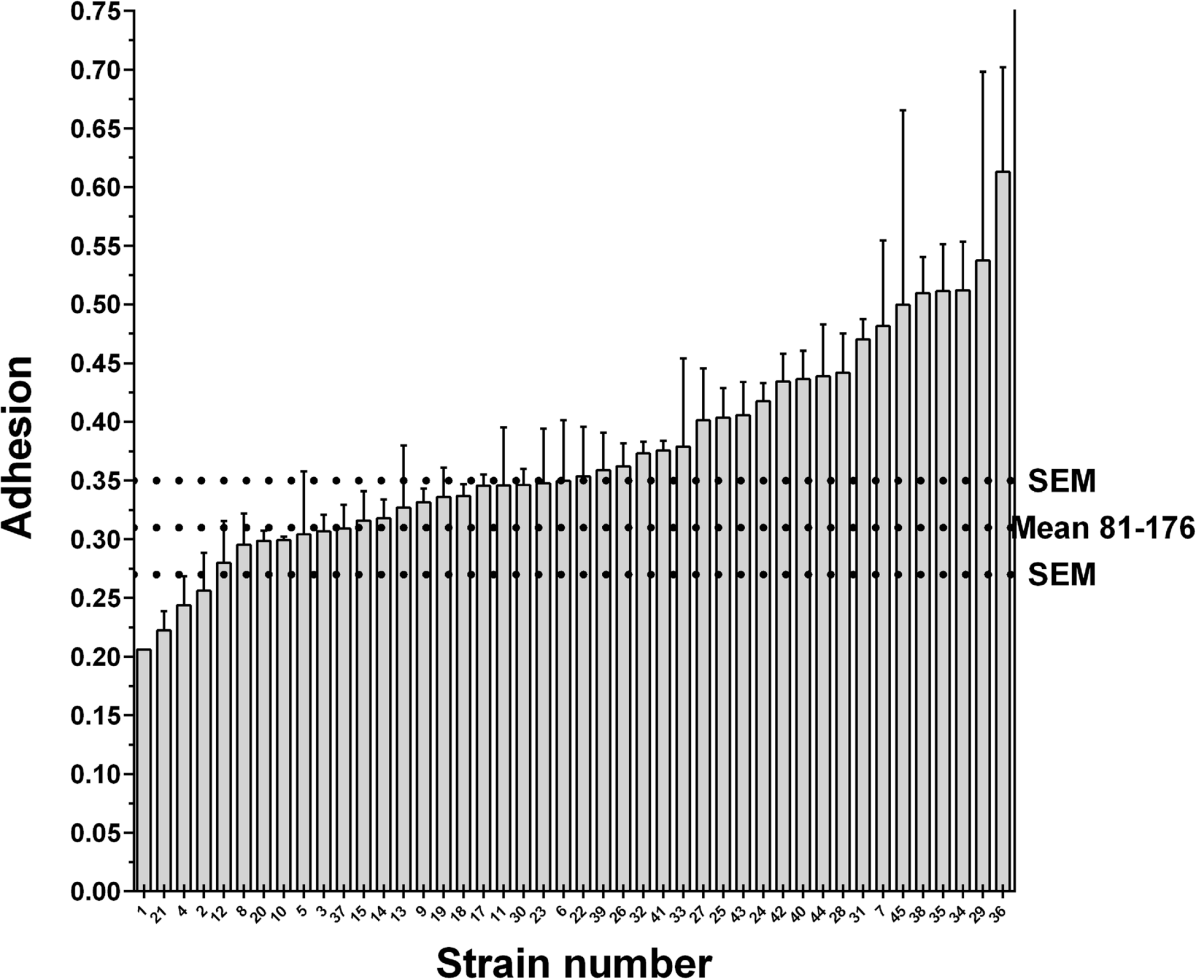
Adhesion to primary caecal chicken cells by the *Campylobacter jejuni* chicken strains. The error bars represent the SEM. The horizontal dashed lines represent the control strain 81–176 and its SEM. Strain results were classified by increasing order. The x axis represents the strains numbered according to their final mean rank value, presented in Table 1. Strains were found with different adhesion properties (Kruskal-Wallis, p < 0.0001). Adhesion is expressed as −1/log (adhered bacteria/initial bacteria used)

**Fig. 4:**
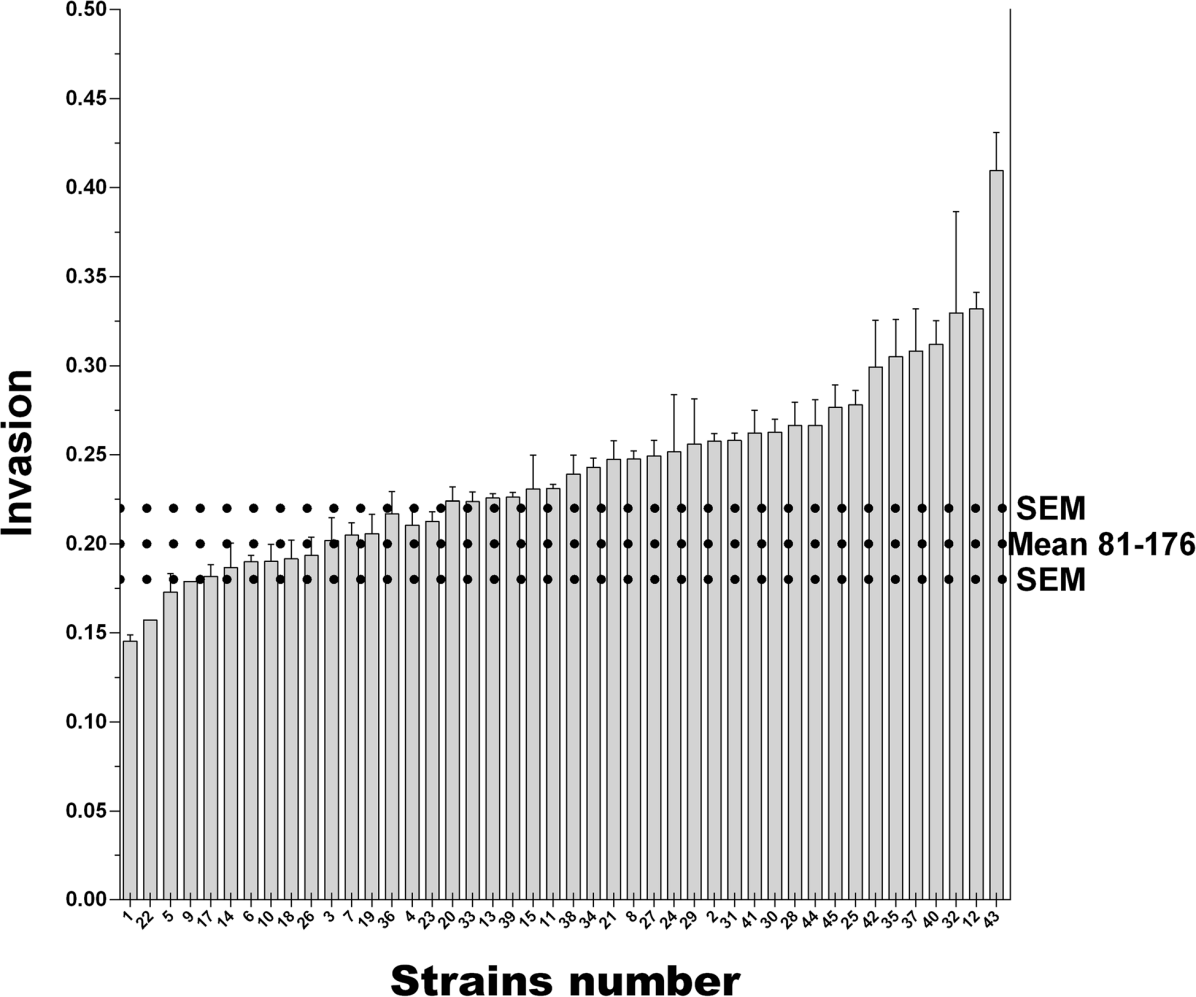
Invasion of primary caecal chicken cells by *Campylobacter jejuni* chicken strains. Invasion levels of primary caecal cells by *C. jejuni* strains using the gentamicin protection assay. The error bars represent the SEM. The horizontal dashed lines represent the control strain 81–176 and its SEM. Strain results were classified by increasing order. The x axis represents the strains numbered according to their final mean rank value, presented in Table 1. Strains were found with different invasion properties (Kruskal-Wallis, p < 0.0001). Invasion is expressed as −1/log (invading bacteria/initial bacteria used)

Strains harbored different autoagglutination (*p* = 0.0083, Fig. 1) as well as chemotaxis (Fig. 2) properties (p < 0.0001). Adhesion and invasion levels (Figs. 3 and 4) to chicken caecal primary cells were also found to be variable (both p < 0.0001). Apart from the obvious differences *in extrema*, no specific group seemed to emerge from these distributions.

**Fig. 5:**
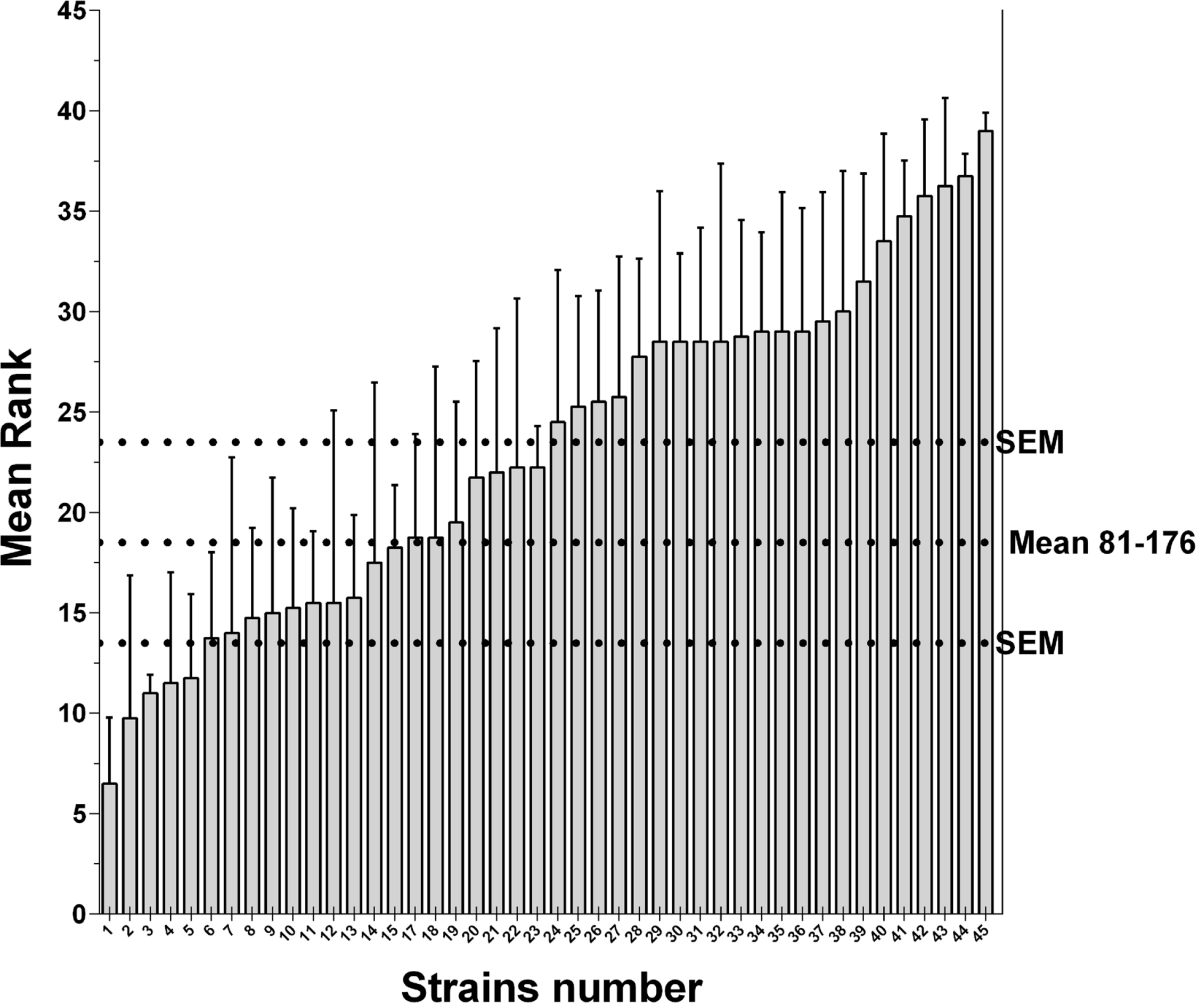
Strain rankings based on their phenotyping properties. For a given strain, the mean rank is based on the phenotypical properties of each strain compared with all others tested in the autoagglutination, chemotaxis, adhesion, and invasion assays. The error bars represent the SEM. On the X axis, strains mentioned in the article are identified. The horizontal dashed lines represent the control strain 81–176 and its SEM. Strains were classified by increasing order. The x axis represents the strains numbered according to their final mean rank value, presented in Table 1. Strains were found to have different mean rank values (Kruskal-Wallis, p < 0.0043)

To take into account all chicken colonization associated phenotypic properties when comparing strains, strains were individually ranked according to their phenotypic properties and the mean rank value for all strains was calculated (Table 1). In our collection, strains displayed different mean rank values, the lowest one being 7 and the highest 39 (p = 0.004, Fig. 5, and Table 1).

We then used k-means partitioning to determine how strains would be objectively grouped, instead of arbitrarily choosing which strain could be considered to present a high or a low competition potential. The strain partitioning was coherent with the calculated mean rank values: group 1 was composed of strains possessing a minimum mean rank of 7 and a maximum mean rank of 18, group 2 was composed of strains possessing a minimum mean rank of 15 and a maximum mean rank of 32, while a third group was composed of strains possessing a minimum mean rank of 25 and a maximum mean rank of 39 (Table 1).

### Chicken colonization competition experiment

Chickens in all groups were negative for *C. jejuni* prior to inoculation but were colonized to the same extent seven days after the co-inoculation (7 PI). For group 1, group 2, and group 3, counts were 8.2 ± 0.7, 8.2 ± 0.7 and 8.2 ± 0.4 log CFU/g of caecal matter ± SEM, respectively. The genes unique to the strains possessing the highest competition potential were systematically detected while the others were not (Table 3).

### Chicken colonization gene content association with the strains’ mean rank

The gene content of the strain possessing the highest mean rank value (A2008a) was compared with the gene content of the strain possessing the lowest mean rank value (D2008b). This comparison established that 20 genes were found to be present only in the highest ranking strain (A2008a) (data not shown). Their presence was assessed in the remaining strains. Of these, eight were present among *C. jejuni* strains that also significantly possessed higher mean rank values than the strains lacking them (Table 4). In the corollary analysis, a total of 27 genes were found present only in the strain (D2008b) possessing the lowest mean rank value (data not shown). Of these genes, 11 were present among *C. jejuni* strains that significantly possessed a low mean rank value compared to the strains lacking these genes (Table 5). This allowed the identification of genes that were associated with strains presenting significantly greater or lower outcompeting capacities.

**Table 4:** Genes associated with *Campylobacter jejuni* strains possessing higher potential competition capacities

**Gene**	**Rank**	**Agg**	**Chm**	**Inv**	**Adh**	**Gene Function**
	**p value**	**p value**	**p value**	**p value**	**p value**	
*ars*C	0.001	0.254	0.007	0.027	0.037	Arsenate reductase
C8J1456	0.005	0.824	0.002	0.033	0.175	Hypothetical protein
C8J1083	0.006	0.788	0.001	0.063	0.172	Hypothetical protein LOS locus
*dms*A	0.010	0.930	0.002	0.085	0.143	Anaerobic dimethyl sulfoxide reductase
*rlo*A	0.021	0.826	0.003	0.085	0.113	type 1 restriction system variant
*HsdS*	0.039	0.857	0.007	0.221	0.279	type 1 restriction system variant
CJE1719	0.039	0.213	0.069	0.025	0.008	Zinc-binding dehydrogenase family variant
C8J0988	0.043	0.514	0.026	0.127	0.013	Hypothetical protein

In the characterized *C. jejuni* strain collection, strains positive by microarray for these genes had higher mean rank values than the negative strains, as assessed by the Mann–Whitney test. An alpha value lower than 0.05 was considered for genes present in strains likely to possess different mean rank values or phenotypical properties, while an alpha value lower than 0.002 was considered for identifying genes that are of greater importance; Phenotypic properties refer to Agg = autoagglutination; Chm = chemotaxis; Inv = invasion; Adh = adhesion.

**Table 5:** Genes associated with *Campylobacter jejuni* strains possessing lower potential competition capacities

**Gene**	**Rank**	**Agg**	**Chm**	**Inv**	**Adh**	**Function**
	**p value**	**p value**	**p value**	**p value**	**p value**	
*lct*P	0.002	0.222	0.018	0.037	0.023	L-lactate permease
CJ1325	0.003	0.538	0.001	0.054	0.112	Putative periplasmic protein
CJE1277	0.004	0.538	0.002	0.037	0.087	Putative glycosyltransferase
CJE0171	0.006	0.796	0.003	0.070	0.049	Putative TonB-dependant outer membrane receptor
CJE1679	0.009	0.436	0.147	0.017	0.011	Methyl-accepting chemotaxis protein
CJ0223	0.010	0.983	0.037	0.002	0.266	Pseudogene, putative IgA protease family protein
*mot*A	0.010	0.920	0.002	0.085	0.143	Flagellar motor proton channel possible variant
CJE1820	0.012	0.885	0.011	0.019	0.039	Putative periplasmic protein
CJE1730	0.021	0.645	0.015	0.068	0.041	Putative permease
*cfr*A	0.030	0.677	0.008	0.218	0.233	Ferric receptor cfrA
CJE1719	0.039	0.213	0.069	0.025	0.008	Zinc-binding dehydrogenase family oxidoreductase variant

In the characterized *C. jejuni* strain collection, strains positive by microarray for these genes had lower mean rank values then the negative strains, as assessed by the Mann–Whitney test. An alpha value lower than 0.05 was considered for genes present in strains likely to possess different mean rank values or phenotypical properties, while an alpha value lower than 0.002 was considered for identifying genes that are of greater importance; Phenotypic properties refer to Agg = autoagglutination; Chm = chemotaxis; Inv = invasion; Adh = adhesion.

### CGF typing

**Fig. 6:**
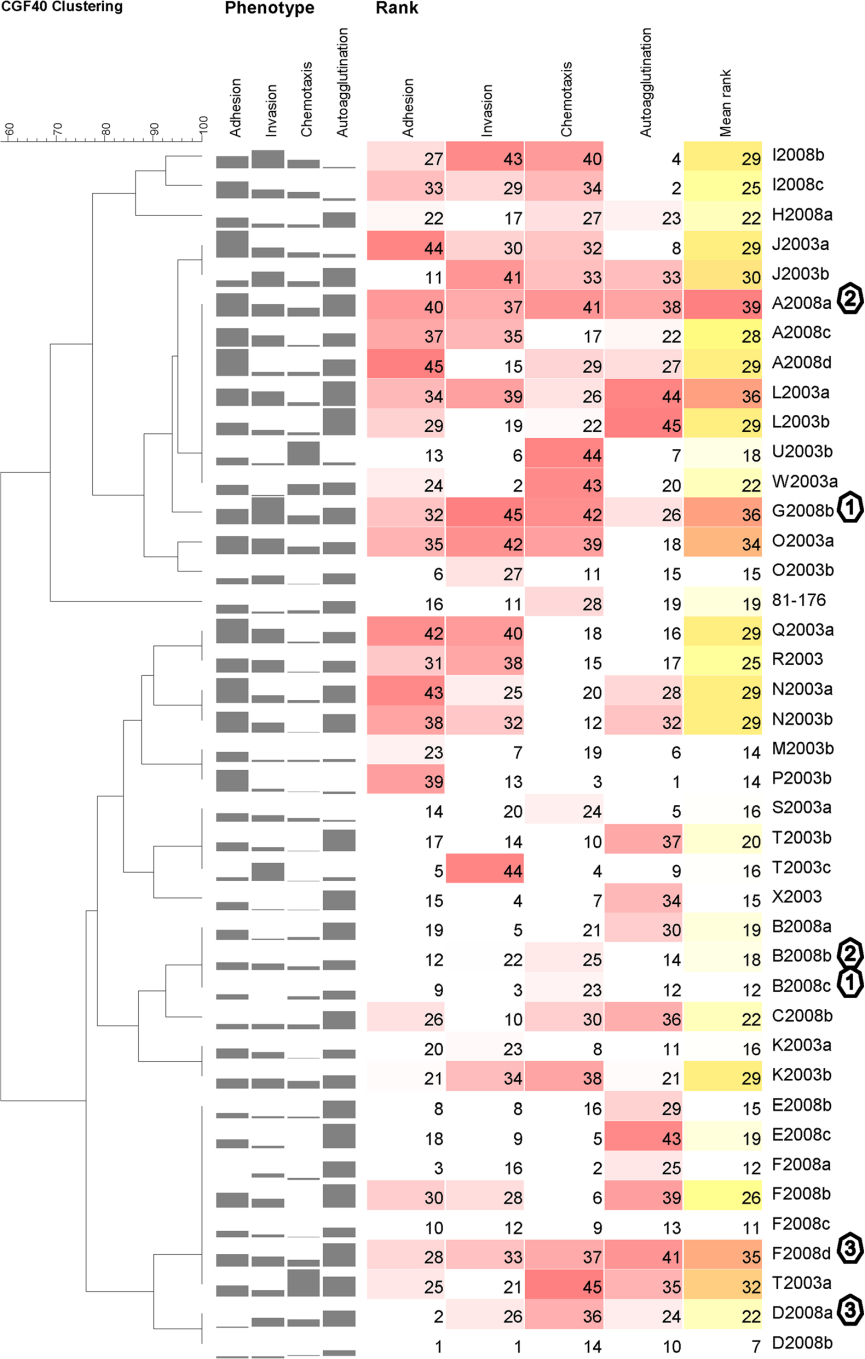
Strain CGF clustering, phenotype, and ranking. CGF data were used to cluster strains. For each strain, an overview of its phenotype is shown. For each property (adhesion, invasion, chemotaxis, and autoagglutination), the higher the bar is, the higher the property was. For each strain, its rank in each phenotypic assay is shown. For each assay, the strain with the lowest property was assigned a rank of 1 and the highest a rank of 45. Mean rank is the mean of all four property rank values. The darker the rank value background is, the higher the value. Strain identification begins with a letter (example **H**2008b) representing the lot origin, followed by the year (example H**2008**b) of sampling and ends with an identification letter (example H2008**b**) to differentiate between strains isolated from the same lot. Strains sharing the same octagon number were the strains co-inoculated to each corresponding chicken group in the chicken colonization competition assay

Out of the 45 strains analyzed in this study, 26 different CGF profiles were observed among the strains in the dataset, including three newly identified fingerprints (Additional file 1 Table S1, and Table S2). Most grouped strains also possessed similar outcompeting potential (Fig. 6).

## Discussion

Various *C. jejuni* strains do not seem able to colonize the chicken gut with the same ability and the extent of this variability was unknown. This study thoroughly characterized chicken *C. jejuni* strains in an attempt to better understand the competitive advantages for chicken caecal colonization.

It has been shown that some phenotypic properties, taken individually, were important for effective chicken colonization [7]. It has also been demonstrated that strains with superior *in vitro* invasion capabilities can outcompete others in chicken colonization assays [10]. In our study, we characterized a collection of chicken *C. jejuni* strains according to 4 important phenotypic properties that may affect colonization. With this characterization, we clearly showed the variability of the autoagglutination, chemotaxis, adhesion, and invasion properties and showed that they were unexpectedly well distibuted among the tested chicken *C. jejuni* strains.

We then proposed a novel classification method that allowed us to integrate all characterized phenotypical properties when comparing sets of strains in order to better estimate their chicken competition potential. K-means partitioning distributed the strains in the 3 groups and was able to clearly separate strains between those that possessed the best or the poorest phenotypic properties mean rank values; strains with high mean rank values were considered as highly competitive while those with low mean rank values were expected to be poorer competitors. This demonstrated that even though all strains were initially recovered from chicken caecal content, they were quite diverse when comparing their competition potential. The strains possessing the highest competition potentials were those that also showed high autoagglutination, chemotaxis, adhesion, and invasion properties.

To corroborate that the classification of the strains according to their mean rank value reflected the chicken outcompeting capacity of the strains, an *in vivo* assay was carried out. Strains from the suspected high, low, and mild competition potential groups were used. This assay showed that the dominant strains were systematically the ones with the highest mean rank values and that these strains are present in the high competition potential group. Indeed strains possessing low mean rank values were not detected in the diluted (10^−3^) chicken caecal content, indicating a difference of at least 4 log CFU/g for colonization.

These results suggested that not all of our strains were equally adapted to chicken colonization and that strains with high mean rank values would likely outcompete others during chicken colonization. The recovered strains with the lowest outcompeting capacities were probably the only ones that the sampled chickens encountered during rearing.

Comparison of the gene content of the best performing to the poorest performing strain allowed the identification of genes in strains possessing different mean rank values and thus different outcompeting capacities. Of these genes, the *ars*C gene was strongly associated with strains (p = 0.001) possessing high mean rank values. This gene was previously identified in a study that compared the gene content of two strains possessing different colonization capacities [19]. In *C. jejuni*, *ars*C is part of an operon conferring resistance to arsenic [20]. Arsenical compounds such as arsanilic acid were used in Canada to control coccidiosis in chicken [21]. Arsenic resistance is variable in *C. jejuni* strains [20]. The exact role of *ars*C in *C. jejuni* chicken colonization remains to be validated. An indirect role for *ars*C in colonization has been proposed for *Yersinia* where arsenate resistance probably participated in the selective emergence of more specialized pig strains but was not directly involved in colonization [22].

This is, to our knowledge, the first report of genes that may be associated with different outcompeting capacities. The exact importance of these genes for competition remains to be further assessed.

The comparison of the CGF profiles obtained in the present study was made with those present in a Canadian database of over 15,000 isolates from human, animal, and environmental sources. The biggest cluster found was 926.2.1 (Additional file 1 Table S1, and Table S2) and all strains in this cluster had higher outcompeting capacities. In the Canadian database, strains from this cluster were isolated from chickens, raccoons, and humans. Further studies that would extend the characterization of strains occurring from different sources are needed to make conclusions about the public health impact of the ability of a given strain to compete for chicken colonization. CGF profiles did not perfectly match outcompeting capacity, further illustrating the disparity that exists between strain genetic and phenotypic typing.

## Conclusions

In this study, a combination of phenotypic and genetic characterization was used in order to identify traits that provide *C. jejuni* strains with a competitive advantage when colonizing the chicken gastrointestinal tract. It clearly demonstrated the extent of the variability of phenotypes found in chicken *C. jejuni* strains. Our results confirm that some *C.jejuni* strains are better competitors than others. Strains with high autoagglutination, chemotaxis, adhesion, and invasion properties were the ones presenting the highest outcompeting capacities. Some genes could be associated with a strain’s outcompeting capacities such as the arsenic resistance gene *ars*C. The role and direct involvement of the identified genes in the mechanism that confers some *C. jejuni* a competitive advantage when colonizing the chicken gut still needs to be assessed in further studies.

## Additional file

Additional file 1: Table S1. Describes, for each typed strains, the presence or absence of all 40 genes tested by PCR; In red are the genes missing from the strains and in green the genes present; The last column indicates the CFG cluster identification according to the Canadian database. **Table S2.** Describes, for each cluster observed in this study, all meta-data available in the Canadian database.
